# NKCS, a Mutant of the NK-2 Peptide, Causes Severe Distortions and Perforations in Bacterial, But Not Human Model Lipid Membranes

**DOI:** 10.3390/molecules20046941

**Published:** 2015-04-16

**Authors:** Corina Ciobanasu, Agnieszka Rzeszutek, Ulrich Kubitscheck, Regine Willumeit

**Affiliations:** 1 Institute for Physical and Theoretical Chemistry, Rheinische Friedrich-Wilhelms-University Bonn, Wegeler Str. 12, 53115 Bonn, Germany; E-Mail: u.kubitscheck@uni-bonn.de; 2 Department of Interdisciplinary Research, Alexandru I. Cuza University, Blvd. Carol I, no. 11, Iaşi RO-700506, Romania; 3 Department of Structural Research on Macromolecules, Institute of Materials Research, Helmholtz-Zentrum Geesthacht, Max-Planck-Str. 1, 21502 Geesthacht, Germany; E-Mail: Agnieszka.Rzeszutek@hzg.de

**Keywords:** antimicrobial peptides, membrane distortion, membrane perforation, phosphatidylethanolamine

## Abstract

NKCS is an improved mutant of the bioactive peptide NK-2, which shows strong activity against *Escherichia coli* and low toxicity towards human cells. The different activity demonstrates the relevance of the physico-chemical nature of the target membrane for the biological effect of this peptide. We studied the effect of this potent antimicrobial peptide on model membranes by activity studies, differential scanning calorimetry, single molecule tracking and tracer efflux experiments. We found that NKCS severely distorted, penetrated and perforated model lipid membranes that resembled bacterial membranes, but not those that were similar to human cell membranes. The interactions of NKCS with phosphatidylethanolamine, which is abundant in bacterial membranes, were especially strong and are probably responsible for its antimicrobial activity.

## 1. Introduction

In recent years the problem of antibiotic resistance has increased dramatically. Modifications of traditional drugs appear to be a short-term solution, since pathogens rapidly adapt their resistance mechanisms to overcome the problem of susceptibility [1]. Thus, the development of new effective antimicrobial agents is urgent. Cationic antimicrobial peptides (AMPs) represent a therapeutic alternative [2,3]. Such peptides have been found in all living organisms ranging from prokaryotes to plants, invertebrates and vertebrates, including human beings [4,5,6,7,8]. In bacteria they are produced as a natural weapon to fight other bacterial cells competing for nutrients in the same environment [9]. In higher organisms they constitute a part of the innate immune system showing direct antimicrobial activities against a broad spectrum of pathogens [10,11] or acting as immunomodulators [12].

Hundreds of antimicrobial peptides have been identified [13], but so far only very few of them have made it into clinical trials or can even be used as new antibiotics, e.g., omiganan and derivatives or SB-006. This is partially due to fact that a kind of “trial and error” approach is used in drug development, because the exact mechanisms of action remained unclear for most active compounds.

In general, AMPs can be divided into two classes: membrane-disruptive and membrane-nondisruptive antibiotics [14,15]. The first group causes permeabilization of bacterial membranes and cell lysis, whereas the latter interacts with specific cellular targets. However, the classification of peptides according to their mode of action is difficult. Molecules with the capability to lyse cell membranes can also exhibit a specificity for cellular targets. Moreover, if an AMP targets strongly the membrane of one group of microorganisms, it may kill cells of another group solely in a nondisruptive way. Still, in many situations the interaction with the cytoplasmic membrane is an important step—either as direct target or as a barrier, which has to be passed. In most cases AMPs interact directly with phospholipids without exploitation of any protein receptors [16,17]. Such lipid-directed mechanisms imply that the composition and architecture of the membrane determines the selectivity, specificity and activity of AMPs [18,19]. Also, certain structural and physicochemical features of AMPs define their antibiotic potential. The required properties are manifold. In general they include a positive net charge, which is responsible for the electrostatic attraction to the negatively charged target cell surface. However, the amount of charged residues can vary significantly and they can be counterbalanced by the presence of ions. Hydrophobicity, which is important for the interactions of peptides with the core of lipid bilayers, is another important requirement, but again it is not necessarily an indication for the potency of an AMP as antimicrobial drug. Last, but not least, flexibility of the peptide chain in terms of its ability to easily bend or kink appears necessary to allow the transition of peptides from their unordered structure in the medium to an ordered conformation induced by a membrane [17,20]. In conclusion, understanding the mechanisms of action and identification of parameters defining the antimicrobial potency of natural peptides will allow to design novel molecules with enhanced activity, based on already existing active structures.

Here, we investigated the interactions of the antimicrobial peptide NKCS with the cytoplasmic membrane of the model Gram-negative bacteria *Escherichia coli*. The primary structure of NKCS is directly related to the well investigated cationic antimicrobial peptide NK-2 [21,22,23,24,25,26], which was derived from the naturally occurring 78-amino acid residue protein NK-lysin [27]. NK-lysin was isolated from porcine small intestine and displays cytotoxic and antimicrobial activity [25]. The sequence of NK-2 has been used as a template to design new derivatives with improved activity and to understand the relevance of structural and physicochemical peptide features such as α-helicity, charge and amphipathicity for the interactions with the membrane of pathogens [28,29]. NKCS was derived from NK-2 by replacement of the cysteine at position 7 by serine, what yielded a more stable peptide, which is resistant to oxidation. Previous circular dichroism experiments revealed that the peptide is randomly coiled in aqueous solution, but adopts an α-helical structure in an hydrophobic environment [30]. Moreover, Monte Carlo simulations suggested that NKCS does not form a rigid straight rod, but rather a helix-hinge-helix structure when binding to membranes [30].

We used artificial membrane systems composed of phosphatidylcholine (PC), phosphatidylethanolamine (PE) and/or phosphatidylglycerol (PG) as targets for NKCS. PC is abundant in membranes of human cells. PE and PG were chosen because they mimic the cytoplasmic membrane of *E. coli* [31]. The effect of NKCS on the lipid phase behavior was investigated by differential scanning calorimetry (DSC). Single molecule tracking techniques and confocal laser scanning microscopy were employed to study the interaction of the peptides with the membranes, membrane penetration and pore-forming capabilities [32,33,34,35,36]. We found that NKCS severely distorted, penetrated and perforated model lipid membranes that resembled bacterial membranes, but not those that were similar to human membranes.

## 2. Results and Discussion

### 2.1. Hemolytic and antibacterial activity

**Figure 1:**
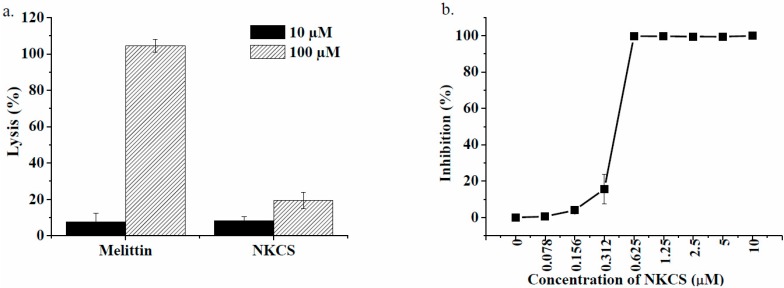
Biological activity of NKCS. (**a**) Hemolytic activity of the nonselective membranolytic peptide melittin and of NKCS; (**b**) Growth inhibition of labeled NKCS on *E. coli*. The activity of the dye-labeled peptides was comparable to the unlabeled species.

The cytotoxicity of NKCS towards eukaryotic cells was investigated using human erythrocytes. NKCS showed a low hemolytic activity, even at a concentration of 100 µM (Figure 1a). This was in clear contrast to melittin, a membranolytic peptide known for its non-selectivity [37], which caused complete lysis of red blood cells at a concentration of 100 µM.

Next, the peptide NKCS was tested against the model Gram-negative bacterium *E. coli*. NKCS appeared very active with a minimal inhibitory concentration (MIC) at 0.6 µM, which was the same as previously reported [30] (Figure 1b).

### 2.2. Differential Scanning Calorimetry

**Figure 2:**
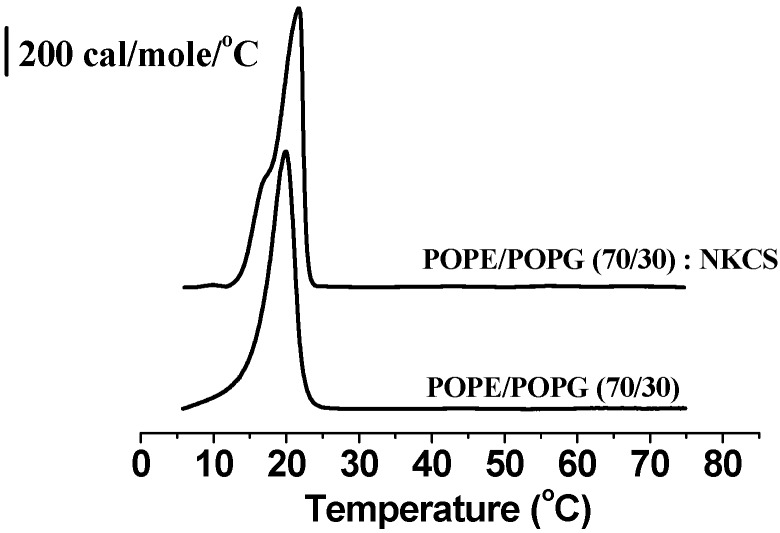
Heat capacity function of liposomes composed of POPE and POPG at a molar ratio of 70:30 without and with NKCS (lipid:peptide molar ratio of 100:1). The curves are vertically shifted for the sake of clarity.

The impact of antibiotic peptides on the phase behavior of phospholipids generally reflects their activity and provides a good hint regarding their mode of action [38,39]. Liposomes mimicking the cytoplasmic membrane of bacteria were composed of POPE and POPG at the ratio 70:30. Despite the fact that POPE can exhibit a hexagonal phase for temperatures above 70 °C, the combination with POPG resulted in vesicles, which remained in a stable lamellar phase in the studied temperature range of 3 °C to 75 °C, as the results of DSC revealed (Figure 2). At 19.9 °C the transition from gel into a liquid crystalline phase was observed. The almost symmetrical peak indicated the good mixing of the phospholipids, and cooperative chain melting. A distinct alteration of the lipid phase behavior was observed when NKCS was added at a lipid:peptide molar ratio of 100:1, which corresponded to a concentration of 65 µM, well above the MIC in the antibacterial activity test discussed above (Figure 1). Now, the phase transition was shifted to a higher temperature, namely 21.7 °C and a distinct shoulder appeared suggesting the separation of phospholipids induced by a strong interaction of the positively charged NKCS. Presumably, the positively charged NKCS interacts stronger with the negatively charged POPG and thus leads to a spatial separation of POPG and POPE.

### 2.3. Diffusion of Single Lipid Tracer Molecules in GUV Membranes

DSC provided a static overview of the peptide-bilayer interactions. Next, we studied the dynamics of the NKCS-membrane system. We started out by quantifying the lipid dynamics within respective model bilayers to obtain a reference for the NKCS dynamics. Fluorescent lipid analogs were inserted into GUVs created by electro-formation at very low molar ratios. The high dilution of the fluorescent tracer molecules made it possible to observe single diffraction limited signals by sensitive fluorescence microscopy well separated from each other, and to follow their diffusion pathways within the membrane by high speed imaging [40]. From the single molecule trajectories the diffusion coefficients of the molecules could be determined. Unfortunately, it was not possible to generate GUVs from pure PE or pure PG by electroformation. Also PE:PG GUVs could not be formed due to their negative charge [26]. Rather, we had to add a certain amount of PC to form stable GUVs. We determined the mobility of the fluorescent lipid tracer TR-DHPE in GUV membranes of three different compositions: GUVs comprising DOPC alone mimicking human erythrocyte membranes, GUVs comprising DOPC/POPE at a molar ratio of 60/40 and GUVs comprising DOPC/POPG at a molar ratio of 70/30 as approximations to bacterial membranes.

**Figure 3:**
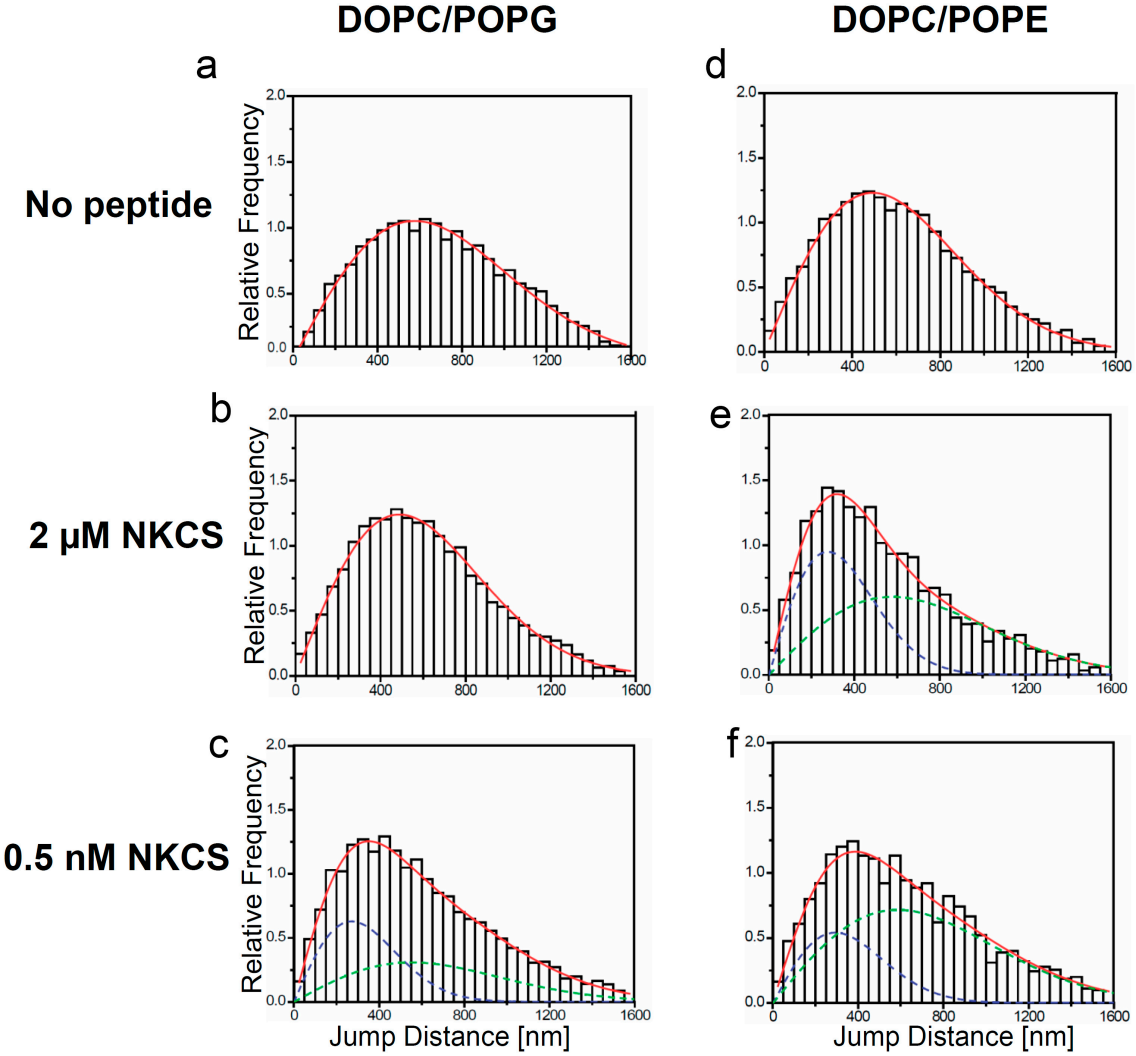
Jump distance distributions for TR-DHPE and NKCS molecules in GUV membranes as determined from single molecule trajectories. The jump distance analysis was performed for TR-DHPE in GUVs prepared from (**a**) DOPC/POPG and (**d**) DOPC/POPE, and for TR-DHPE in presence of 2 µM unlabeled NKCS in GUVs prepared from (**b**) DOPC/POPG and (**e**) DOPC/POPE. The lower panel shows the jump distance analysis for 0.5 nM Dy647-NKCS on GUVs formed by (**c**) DOPC/POPG and (**f**) DOPC/POPE, respectively, revealing the peptide mobility. All full red lines indicate the fitting results according to Equation (1) with one (a, b, d) or two (c, e, f) mobility terms. The green and blue dashed lines in (c, e, f) show separately the low (blue) and high (green) mobility fraction, when two diffusion terms were required to satisfactorily fit the data.

Extensive single molecule tracking experiments showed that the lipid tracer diffused with a diffusion coefficient of about 5 ± 0.4 µm^2^/s within membranes made from DOPC alone and within membranes made from DOPC/POPG. The mobility was reduced to 3.7 ± 0.2 µm^2^/s in a membrane comprising DOPC/POPE (see Figure 3a,d, and Table 1). Accordingly, DOPC and DOPC/POPG membranes have a comparable viscosity, but that of DOPC/POPE-GUVs was higher by approximately 30%. This was not surprising since it is known that PE establishes more intermolecular hydrogen bonds yielding a higher viscosity [41].

**Table 1:** Mobility of lipid tracers (grey background) and NKCS (white background) within GUV membranes of different composition determined by single molecule tracking. Two rows of numbers in one field mean: Upper rows are the diffusion constants, lower rows are the fraction of this mobility component with respect to the whole system.

Probe Molecule	D_SPT_ [µm^2^/s]				
	DOPC	DOPC/POPG		DOPC/POPE	
TR-DHPE	5.3 ± 0.4	5.0 ± 0.2		3.7 ± 0.2	
TR-DHPE	5.4 ± 0.2	3.6 ± 0.3		5.0 ± 0.6	1.2 ± 0.1
+2 µM NKCS				0.57 ± 0.02	0.43 ± 0.02
NKCS	5.6 ± 0.1 *	4.9 ± 0.3	1.1 ± 0.1	5.2 ± 0.7	1.3 ± 0.3
		0.59 ± 0.03	0.41 ± 0.03	0.7 ± 0.07	0.3 ± 0.08

* A peptide concentration of 1 nM instead of 0.5 nM was used in order to obtain a sufficient number of single molecule tracks.

### 2.4. Modification of the GUV Membrane Dynamics by NKCS

The effect of NKCS on the bilayer viscosity itself was studied by measuring the mobility of the TR-DHPE lipid tracers in the presence of the unlabeled NKCS at a concentration of 2 µM. This concentration was well above the MIC to make sure that the peptide is on, respectively in membrane (Figure 3). Now, the tracer mobility in GUVs made from DOPC was not affected by the presence of the peptides (Table 1). This was expected in view of the results of the hemolysis experiments, which indicated that NKCS does not strongly interact with erythrocyte-like membranes. In stark contrast to this, the tracer mobility in membranes comprising DOPC/POPG was reduced by approximately 30% with respect to the undisturbed membrane. A further effect was observed when the peptides were added to the GUVs comprising DOPC/POPE. In these model membranes the lipid tracer mobility now displayed a bimodal distribution. One fraction of the lipids showed a higher mobility compared to the peptide-free membrane, *i.e.*, the diffusion coefficient increased from 3.7 µm^2^/s to more than 5 µm^2^/s. This number was close to the value of pure DOPC membranes. The other fraction was strongly slowed down to 1.3 µm^2^/s. These findings indicated the occurrence of a peptide-induced lipid phase separation in DOPC/POPE membranes. Probably the peptides were concentrated in a POPE-rich phase, which displayed strong peptide-POPE interactions reducing the tracer mobility, whereas the DOPC-rich domains remained practically unaffected by the peptides, and hence showed the higher mobility of DOPC-GUVs as discussed above.

### 2.5. Single NKCS Peptides Traced within GUV Membranes

To examine the behavior of NKCS peptides within the GUV membranes Dy647-labeled NKCS was used as probe in single molecule tracking experiments. GUVs were incubated for 30 min with 0.5 nM of the fluorescent peptides. During this time the peptides bound to the GUV membranes. As described above for the lipid tracer molecules the diffusion pathways of single Dy647-peptides in the GUV membrane could be recorded and analyzed. As in the previous experiments the mobility characteristics were quantified by jump distance histograms.

In GUVs formed from DOPC alone we detected only very rarely single peptide signals. This demonstrated again the weak interaction between the peptides and PC-lipids in DOPC membranes. In order to register enough events for jump distance histograms the NKCS concentration was increased to 1 nM. Then, the peptide displayed a mobility of 5.6 µm^2^/s (Table 1). This value corresponded well to the mobility of the lipid tracers, and suggested that there were no special interactions between the peptides and PC membranes. Probably, in rare cases the peptides loosely attached to and diffused together with single lipid molecules, but did not show distinct and long-lasting interactions, and certainly no incorporation into the bilayer. A similar outcome was previously obtained for TAT peptides, which was interpreted as a “floating” of the peptides on the membrane surface [32].

The situation was strikingly different for GUVs made from DOPC/POPG and DOPC/POPE. Here, characteristic jump distance histograms exhibiting *two* mobility components were obtained. However, an important difference was observed in the diffusion constants obtained for the different lipid mixtures. In the case of the PG-containing vesicles one diffusion constant was close to the lipid tracer mobility of the undisturbed system, namely 4.9 µm^2^/s (as compared to 5.0 µm^2^/s of the tracers in DOPC/POPG), but the second diffusion constant was strongly reduced to 1.1 µm^2^/s (Figure 3c). Thus it was even significantly smaller than the lipid tracer mobility of the system disturbed by the high NKCS concentration. For PE-containing vesicles the diffusion constant of the peptides was in the range of the two lipid tracer diffusion constants presented above (Figure 3e,f).

### 2.6. CLSM Imaging of NKCS Peptide-GUV Interaction

The interaction of a fluorescently labeled peptide such as Dy647-NKCS with model lipid membranes can directly be visualized using confocal laser scanning microscopy [32,33,35]. To this end, GUVs prepared from DOPC, DOPC/POPG and DOPC/POPE were incubated with 2 µM of Dy647-labeled NKCS. To monitor the integrity of the GUV membranes large FITC-labeled dextran molecules with a molecular mass of 40 kDa, which would diffuse into the GUVs in case of major membrane leakages, were added to the external solution.

In order to examine putative membrane pores, we prepared three batches of GUVs, and enclosed fluorescent tracer molecules of increasing molecular mass, namely Alexa Fluor 488 (0.64 kDa), 3 kDa AlexaFluor 546-dextran and 70 kDa AlexaFluor 546-dextran, within these batches, respectively. In case pores were formed upon peptide addition a release of the enclosed molecules into the buffer lead to a loss of the initial internal GUV fluorescence, and was monitored as a function of time. In this manner we obtained information about the possible existence and size of AMP-induced membrane pores.

**Figure 4:**
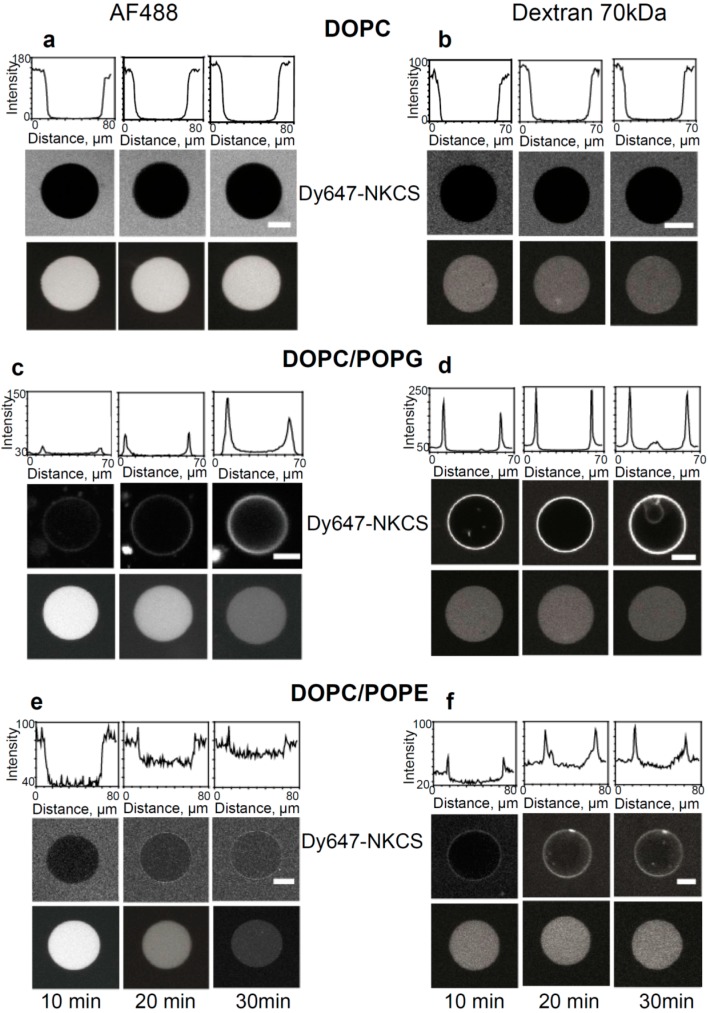
Internalization and tracer release induced by the NKCS peptide in GUVs. (**a**,**b**) Interaction of NKCS with GUVs prepared from DOPC. The middle panel shows neither accumulation nor internalization of the peptide on the GUV membrane. The top panel quantifies the fluorescence intensity along a horizontal line through the center of the GUV. The lower panel demonstrates that (a) AF488 and (b) 70 kDa dextran-Alexa Fluor 546 remained contained within the GUV. (**c**,**d**) Binding of Dy647-NKCS to the membrane formed by DOPC/POPG. Peptide internalization was indicated by the non-zero fluorescence in the GUV interior, as quantified in the upper panel showing the fluorescence intensity along a horizontal line through the center of the GUV. The lower panel demonstrates that there was leakage of (b) AF488 but not of (d) 70 kDa dextran-Alexa Fluor 546 out of the GUV. (c) Interaction of NKCS with GUVs prepared from DOPC/DOPE. The top panel quantifies the fluorescence intensity along a horizontal line through the center of the GUV. The middle panel shows the accumulation of the peptide in the membrane. But additionally over time a portion of the NKCS peptide is crossing the membrane and can be found inside the vesicle. The lower panel demonstrates that there was leakage of (**e**) AF488 but not of (**f**) 70 kDa dextran-Alexa Fluor 546 out of the GUV. All scale bars, 20 µm.

As expected, for GUVs made solely from DOPC confocal microscopy did not show any binding of NKCS to the GUV surfaces. Also, we did not observe peptide internalization into the GUVs, nor efflux of any of the tracers (Figure 4a,b).

The results were different for GUVs prepared from DOPC/POPE. Here, a reduced peptide binding to the GUV was seen, but the influx of the labeled peptides was more efficient and faster than for DOPC/POPG membranes (see Figure 4e,f). The efflux experiments demonstrated the rapid and complete release of Alexa 488 and 3 kDa Alexa546-dextran molecules within 30 min in the presence of NKCS (see Figure S11c for 3 kDa Alexa Fluor 546-dextran). Obviously, again the pores induced by peptides were small, since large dextran molecules with a molecular mass of 70 kDa were not released from the GUVs. The results of all confocal experiments were summarized in Table 2.

**Table 2:** Characteristics of NKCS peptide interaction with GUVs of different membrane composition loaded with tracer molecules of different size.

	DOPC	DOPC/POPG	DOPC/POPE
Peptide influx	−	+	+
AF488 efflux	−	+	+
AF546 Dextran 3 kDa efflux	−	−	+
AF546 Dextran 70 kDa efflux	−	−	−

### 2.7. Discussion

In previous studies the peptide NKCS was shown to be very active against both Gram-negative and Gram-positive bacteria with insignificant toxicity towards human cells [28,30]. These biological characteristics make it an interesting and potent candidate as a novel antibacterial agent, and suggest it as a model for understanding AMP interaction with bacterial membranes. In this study we approached this question with a range of biophysical techniques.

NKCS showed low toxicity toward human erythrocytes. This observation was in good agreement with the result of the CLSM experiment, in which GUVs prepared from DOPC were used to mimic the membrane of human red blood cells, but upon incubation with NKCS there was no peptide accumulation on the membrane. Also, the peptides neither translocate into these GUVs nor did induce effect any efflux of tracer molecules. Finally, single molecule tracking of NKCS on DOPC membranes yielded diffusion coefficients, which were comparable to those of lipid tracer molecules. This indicated that only sporadically and for short periods, the peptides were non-specifically attached to the lipid head groups in the membrane. All this demonstrated that no significant interactions occurred with DOPC and implicitly with model erythrocyte membranes. This conclusion corroborated the results of Andrä *et al.* [28], who did not detect any interactions between NKCS and PC bilayers using circular dichroism and fluorescence resonance energy transfer.

Quite different observations were made, when model lipid membranes inspired by the bacterial system were studied. Measurements of the MIC of NKCS against the Gram-negative bacterium *E. coli* showed that NKCS has a significant antibacterial activity. According to Monte Carlo simulations, upon the membrane interaction NKCS resembles two α-helices connected by a flexible region [30].

Calorimetric studies of POPE and POPG at a molar ratio of 70:30 indicated that NKCS interacted strongly with model bacterial membranes. Without NKCS both lipid components were well mixed and did not form any domains. Upon NKCS addition, however, two different fractions appeared. This indicated that the cationic peptide induced the formation of lipid-peptide domains. The shoulder, which appeared in the thermogram upon addition of NKCS should be attributed to the binding of the peptide to the lipid head groups. Since the intercalation of the peptide between the head groups presumably leads to more space for the acyl chains it can be assumed that the acyl chain melting temperature (*T_m_*) of lipids bound to NKCS is lower than that of the homogenous lipid mixture. Noteworthy, this experiment was performed at lipid:peptide molar ratio of 100:1, which corresponded to a concentration well above the MIC.

To get further insights into the effects of NKCS onto bacterial membranes we employed two bilayer model systems, namely GUVs formed from DOPC/POPE (molar ratio, 60/40), and DOPC/POPG (molar ratio of 70/30). A certain fraction of PC had to be included into the lipid mixture, because pure POPE or POPG did not form GUVs by electro-formation. As mentioned above, there were no special interactions between the peptides and DOPC membranes. In GUVs formed from DOPC alone we detected only very rarely single peptide signals and the value of diffusion coefficient for NKCS on DOPC membranes was the same as for lipids in membrane.

Both lipid and peptide dynamics were studied extensively in these systems. As a reference we determined the diffusion coefficients of lipid tracers in the model membranes. The dynamics in the DOPC/POPG membrane was comparable to that of pure DOPC, 5.0 µm^2^/s, whereas the membrane comprising DOPC/POPE showed a lower mobility, 3.7 µm^2^/s. The reason for this difference could be that the lipids DOPC and POPG, which are at room temperature both in the liquid crystalline phase, form very homogeneous and relatively stable mixed GUVs. Both lipids prefer structures with little or positive curvature. Because the low concentration of POPG in comparison to the neutral PC, stress induced by curvature does not play a role and the lipids can freely diffuse. In contrast to POPG, POPE has a small head group thus having the tendency to form inverse curvatures. Furthermore, POPE is slightly negatively charged [26], it exhibits a gel phase at room temperature and the concentration summed up to almost half of the lipid population. Therefore a reduction of lipid mobility was likely, especially when considering the demonstrated existence of a trend to intermolecular hydrogen bonds [41]. We could assume that we did not have very homogenous GUVs in a somewhat meta-stable situation.

Upon the addition of 2 µM NKCS to the solution in DOPC/POPG-vesicles the diffusion constant of the lipid tracer was reduced to 3.6–3.9 µm^2^/s. This could be explained by a strong, probably charge-driven interaction of the peptides with the lipids. Probably we observed the motion of small peptide-linked lipid clusters, presumably of transient nature. However, looking at the mobility of the NKCS peptides—at a much lower concentration—we observed two separate NKCS diffusion constants in the same membrane. We assumed that one constant was due to peptides, which were loosely interacting with the lipid head groups and “floating” on the membrane. Such a behavior was previously observed for positively charged TAT peptides on GUV membranes [32]. The second diffusion showed a striking, about 4-fold reduction in mobility. This suggested a strong interaction of the NKCS peptides with membrane components, *i.e.*, a strong binding to a larger number of lipid molecules. Probably distinct NKCS-POPG clusters were formed, in which the contained molecules diffused together.

Interestingly, for NKCS peptides diffusing on GUVs made from DOPC/POPE we observed a comparable mobility behavior as before for the lipid tracers, when the GUVs were bathed in 2 µM NKCS. Two mobility components were seen for NKCS exhibiting diffusion coefficients of 5.2 and 1.3 µm^2^/s, almost as before for the tracers (5.0 and 1.2 µm^2^/s). 

Still, we did not expect that the extremely low concentration of NKCS molecules (0.5 nM) was capable of causing a significant phase separation, as it was obviously achieved at higher concentrations (65 µM, see Figure 2). Rather, we concluded that the same effect as in the case of the DOPC/POPG-GUVs was responsible for the two mobility fractions: a certain fraction of peptides was “floating” on the bilayer, and another fraction was tightly bound to a major number of lipids, which drifted with a low mobility in the membrane.

Most interestingly, however, tracer efflux experiments clearly demonstrated that NKCS induced the efflux of small tracer molecules out of GUVs. Obviously, NKCS, which adopts an α-helical but bent conformation leading to a partial intercalation into the membrane, formed small lesions in the examined model bilayers. The effect was stronger for PE-containing vesicles than for PG-containing GUVs and could again be explained by the existence of POPE-rich microdomains in the membranes of the DOPC/POPE GUVs.

Altogether, both lipids PG and PE seem to be important for NKCS peptide activity. While in DOPC/POPG model membranes the peptide showed a stronger binding due to the electrostatic attraction, in DOPC/POPE membranes, we observed a reduced peptide binding but the influx of the labeled peptides was more efficient and faster than for DOPC/POPG GUVs. At concentrations well above the MIC, NKCS modifies also the biophysical properties of the model membrane. The tracer mobility in DOPC/POPG membranes was reduced by approximately 30% in presence of 2 µM unlabeled peptide. A different effect was observed when the peptides were added to DOPC/POPE model membranes. In this case, the lipid tracer mobility displayed a bimodal distribution with a diffusion coefficient close to the value of pure DOPC membranes and the other diffusion coefficient strongly slowed down. These observations indicated the occurrence of a peptide-induced lipid phase separation in DOPC/POPE membranes. Most likely, in the antibacterial activity of NKCS, the role of PG is to promote the accumulation of the peptide on the membrane and PE induces the pore formation or micellization and finally the destruction of bacterial membrane.

The comparison of the presented results with our previous data on TAT peptides suggests a quite different mode of action for NKCS than for TAT. For TAT, we observed only the mentioned floating of the peptides on the membrane surface. TAT peptides were loosely attached to the head group region of the lipid bilayer, but at higher concentrations they massively accumulated on the membrane, and destroyed it locally probably by creating strong local electric fields due to their positive charge. This process was proposed by molecular dynamics simulations of TAT peptide translocation [42]. Probably the internalization at thinned and destabilized membrane patches led simultaneously to the formation of transient pores. NKCS, in contrast to TAT, showed a distinct attachment to PG/PE in GUV membranes. The peptides partly floated, but partly showed a strongly reduced mobility indicating the occurrence of distinct peptide-lipid clusters, which presumably also distorted the existing membrane structure. This effect is probably enhanced when lipids have a small head group, like PE. This will increase the trend to the formation of inverted micelles, in which the lipid head groups surround the peptides and form star-like structures. Such micelles then destroy the membrane integrity and can translocate into the membrane-enclosed space—a process that is accompanied by the creation of membrane lesions. In bacterial membranes this is certainly a lethal process.

## 3. Experimental Section

### 3.1. Peptides

NKCS with the amino acid sequence KILRGVSKKIMRTFLRRISKDILTGKK has a molecular mass of 3186 and a net charge of +9 [28]. Unlabeled NKCS and NKCS coupled to fluorescent Dy647 were synthesized by Biosyntan (Berlin, Germany) in purity >95%. The dye was linked to the C-terminus of the peptides via an additional cysteine. Peptide homogeneity was confirmed by MALDI-TOF (Shimadzu, Berlin, Germany) and analytical RP-HPLC (Shimadzu) performed by the manufacturer. The peptides were synthesized with an amidated C-terminus. Melittin, used as a control peptide in the hemolytic assay, was purchased from Sigma-Aldrich (Munich, Germany) with 99% purity. All peptides were stored at −20 °C. Directly before use they were dissolved in double distilled water to a final concentration of 1 mM. Between the experiments all peptide solutions were kept at −20 °C.

### 3.2. Lipids, Dyes and Tracers

The phospholipids 1-palmitoyl-2-oleoyl-*sn*-glycero-3-phosphoethanolamine (POPE), 1-palmitoyl-2-oleoyl-*sn*-glycero-3-[phospho-*rac*-(1-glycerol)] (POPG) and 1,2-dioleoyl-*sn*-glycero-3-phosphocholine (DOPC) were obtained from Avanti Polar Lipids (Alabaster, AL, USA). The lipids were stored air-tight at −20 °C. Fluorescent dextrans and 1,2-dihexadecanoyl-*sn*-glycero-3-phosphoethanolamine labeled with Texas Red (TR-DHPE), fluorescein isothiocyanate (FITC), AlexaFluor 488 and AlexaFluor 546, were obtained from Invitrogen GmbH (Karlsruhe, Germany).

### 3.3. Preparation of Multilamellar Phospholipid Vesicles

POPE and POPG were dissolved in chloroform/methanol (4/1, v/v) (Merck, Darmstadt, Germany) and mixed to obtain a final molar ratio 70:30. The organic solvent was removed by a constant nitrogen stream, and the resulting lipid film was dried overnight in vacuum at 40 °C. Shortly before experiments the lipid films were hydrated with sodium phosphate buffer (10 mM, pH 7.0) and incubated for 2 h at 28 °C with vortexing (1 min) repeated every 30 min. The samples were cooled down to room temperature and equilibrated for 30 min. This procedure resulted in the formation of multilamellar vesicles (MLV).

### 3.4. Preparation of Giant Unilamellar Vesicles

Giant unilamellar vesicles (GUVs) were created by electro-formation as previously described [32]. For GUV production the following lipid mixtures were used: pure DOPC, DOPC/POPG in a 70/30 and DOPC/POPE in a 60/40 molar ratio. 30 µL of the respective lipid mixture in chloroform were deposited on an indium tin oxide (ITO) coated cover slip (SPI Supplies, West Chester, PA, USA), which was then dried in a desiccator in vacuum. The dried lipid film was hydrated with 250 mM sucrose (Sigma-Aldrich, Schnelldorf, Germany) in double distilled water. For the dye leakage experiments the GUVs were prepared in sucrose solutions containing one of the fluorescent tracers tested (AlexaFluor 546-maleimide, 3 or 70 kDa AF546-dextran, respectively) at a concentration of 5 µM. Vesicles in the bulk solution were transferred into a microscope observation chamber (MatTek, Ashland, MA, USA) containing 250 mM glucose solution or 100 mM NaCl in a 150 mM glucose solution. The density difference caused GUV sedimentation, so that they could be examined at the bottom of the sample chamber by inverted fluorescence microscopy.

### 3.5. Hemolysis

Peptide toxicity to eukaryotic cells was measured using human erythrocytes. Fresh blood (maximum storage time was two days) was centrifuged for three minutes at 2000 rpm and the supernatant was discarded. The pellet was washed three times with phosphate buffered saline (PBS: 10 mM sodium phosphate, 2.7 mM potassium chloride, 137 mM sodium chloride, pH 7.4 from Merck. Erythrocytes were resuspended and diluted in MES buffered saline (20 mM morpholinoethanesulfonic acid, 140 mM NaCl, pH 5.5 from Merck) until 20 µL of this suspension added to 980 µL of water gave the absorbance of 1.4 at the wavelength of 412 nm, which was equal to 5 × 10^8^ cells per mL. The peptides were diluted in MES buffer to desired concentrations. Subsequently 20 µL of erythrocytes suspension were added to 80 µL. As controls, erythrocytes were mixed with MES buffer (spontaneous lysis) and double distilled water (maximal lysis). After 30 min of incubation at 37 °C samples were transferred to an ice bath and MES buffer (900 µL) was added. The specimens were centrifuged (10 min, 4 °C, 2000 rpm) and the concentration of released hemoglobin was measured at 412 nm using a Genesys 10 UV spectrophotometer (Thermo Electron Scientific Instruments, Madison, WI, USA).

### 3.6. Antibacterial Assay

Peptide activity was determined using the microdilution method. *Escherichia coli* K12 strain (ATCC 23716) was obtained from the German Collection of Microorganisms and Cell Cultures (DSMZ, Braunschweig, Germany). Series of two-fold dilutions of peptides ranging from 10 µM to 0.1 µM were prepared. The bacteria were cultivated in Luria-Bertani medium (Becton Dickinson, Heidelberg, Germany) at 37 °C while shaking at 160 rpm to reach the logarithmic phase. Aliquots of the cell suspension (10 µL) were added to microtiter plate wells containing 90 µL of peptide solution. The final concentration of bacteria in a well was 1000 colony forming units (CFU) per mL. The plates were incubated for 18 h at 37 °C and the optical density was measured at 620 nm with a microplate reader (Tecan, Crailsheim, Germany). The minimal inhibitory concentration (MIC) was defined as the lowest peptide concentration resulting in the complete suppression of bacterial growth.

### 3.7. Differential Scanning Calorimetry (DSC)

Aqueous suspensions of POPE/POPG liposomes were prepared as described above with a final phospholipid concentration of 6.5 mM. To obtain homogenous large unilamellar vesicles (LUV) the dispersion was passed 17 times through a polycarbonate membrane with 100 nm pores using a liposome extruder (Avanti Polar Lipids). Subsequently NKCS was added in a lipid:peptide molar ratio of 100:1 (corresponding to 65 µM) and the sample was degassed for 15 min. Calorimetric experiments were performed with a MicroCal VP scanning calorimeter (MicroCal, Northhampton, MA, USA) with a heating scan rate of 0.5 °C/min. Heating curves were measured in the temperature interval from 3 °C to 75 °C. Before each scan the lipid dispersions were equilibrated in the calorimetric cell for 15 min at 10 °C. After the first scan the samples were cooled down and rescanned to check the reproducibility of thermograms. The data were analyzed using Origin 8.0 (OriginLab Corp., Northampton, MA, USA).

### 3.8. Single-Molecule Microscopy

Single molecule tracking experiments were performed to visualize single-molecule trajectories of fluorescently labeled lipid tracer molecules (TR-DHPE) and NKCS peptides as described in [32,33]. All experiments were done at 19 °C using an inverted wide-field single-molecule microscope based on an Axiovert 200TV (Carl Zeiss, Jena, Germany) equipped with a 63X NA 1.2 water immersion objective lens and a four-fold magnifier in front of the electron-multiplying (EM) CCD camera (iXon BI DV-860, pixel size of 24 × 24 μm^2^, Andor Technologies, Belfast, UK). An integration time of 33 ms at a frame rate of 30 Hz was chosen for both lipid and peptide imaging. In all experiments we focused onto the upper surface of freshly prepared GUVs, and for each experimental condition three independent experiments comprising 15 movies of 1000 frames each were performed. Finally, all trajectory data of respective experiments were pooled.

### 3.9. Jump Distance Analysis

The movies acquired by the sensitive and fast EMCCD directly showed the trajectories of the fluorescently labeled probe molecules within the top membrane of GUVs. Identification and tracking of single molecule signals was performed by the commercial software Diatrack 3.02 (Semasopht, Chavannes, Switzerland) yielding large numbers of single molecule trajectories. Further data processing was accomplished by ImageJ [43] and Origin 8.0 as previously described [33]. The distances *r* between the positions of single lipid analogs, respectively single peptides, in a trajectory observed in successive frames, which were acquired with a cycle time *t*, were pooled into frequency distributions. These were theoretically described by the probability distribution *p'(r,t) dr* given in Equation (1), which contained contributions from two subspecies 1 and 2 with different diffusion coefficients D_1_ and D_2_ occurring with fractional weights f_1_ and f_2_, respectively:

(1) $$p^{\prime }(r,t)dr=\sum_{j=1}^{N}\frac{Mf_{j}}{2D_{j}t}e^{-r^{2}/4D_{j}t}rdr$$

where M designated the total number of jumps in the analysis, and N = 1 or 2 in this study.

### 3.10. Confocal Microscopy

GUV formation and NKCS peptide interaction with GUV membranes was examined at room temperature using a confocal laser scanning microscope (LSM 510 Meta, Carl Zeiss), which was equipped with a plan apochromat 63X objective lens. AlexaFluor 488 and FITC were excited using the 488-nm line of an Argon ion laser, AlexaFluor 546 and DHPE-TR by the 543 nm line of a HeNe laser, and Dy647 by a HeNe laser emitting at 633 nm. The glucose solution for the GUVs examined in the confocal microscope contained 10 µM of a 40 kDa FITC-dextran for monitoring GUV integrity and stability.

## 4. Conclusions

Calorimetric and fluorescence microscopy studies showed that NKCS severely distorts, penetrates and perforates lipid membranes that mimic bacterial membranes. In contrast, the peptide does not interact with model membranes prepared from DOPC, which are similar to human membranes. Upon the addition of NKCS to DOPC/POPG or DOPC/POPE-vesicles the diffusion constant of the lipid tracer and also of peptides was reduced suggesting a strong interaction of the NKCS with membrane lipids. Tracer efflux experiments also clearly demonstrated that NKCS induced the outflow of small tracer molecules out of GUVs and the effect was stronger for PE-containing vesicles than for PG-containing GUVs. The interactions of NKCS with phosphatidylethanolamine were especially strong and are possibly the main driving force for antimicrobial activity.

## Supplementary Materials

Supplementary materials can be accessed at: http://www.mdpi.com/1420-3049/20/04/6941/s1.
