# Supplementary material for: NKCS, a Mutant of the NK-2 Peptide, Causes Severe Distortions and Perforations in Bacterial, But Not Human Model Lipid Membranes
Source: Molecules. 2015 Apr 16;20(4):6941–58. doi: 10.3390/molecules20046941 (PMC6272639; doi:10.3390/molecules20046941)
Supplement: Supplementary file 1 [file molecules-20-06941-s001.pdf]

Supplemental Material

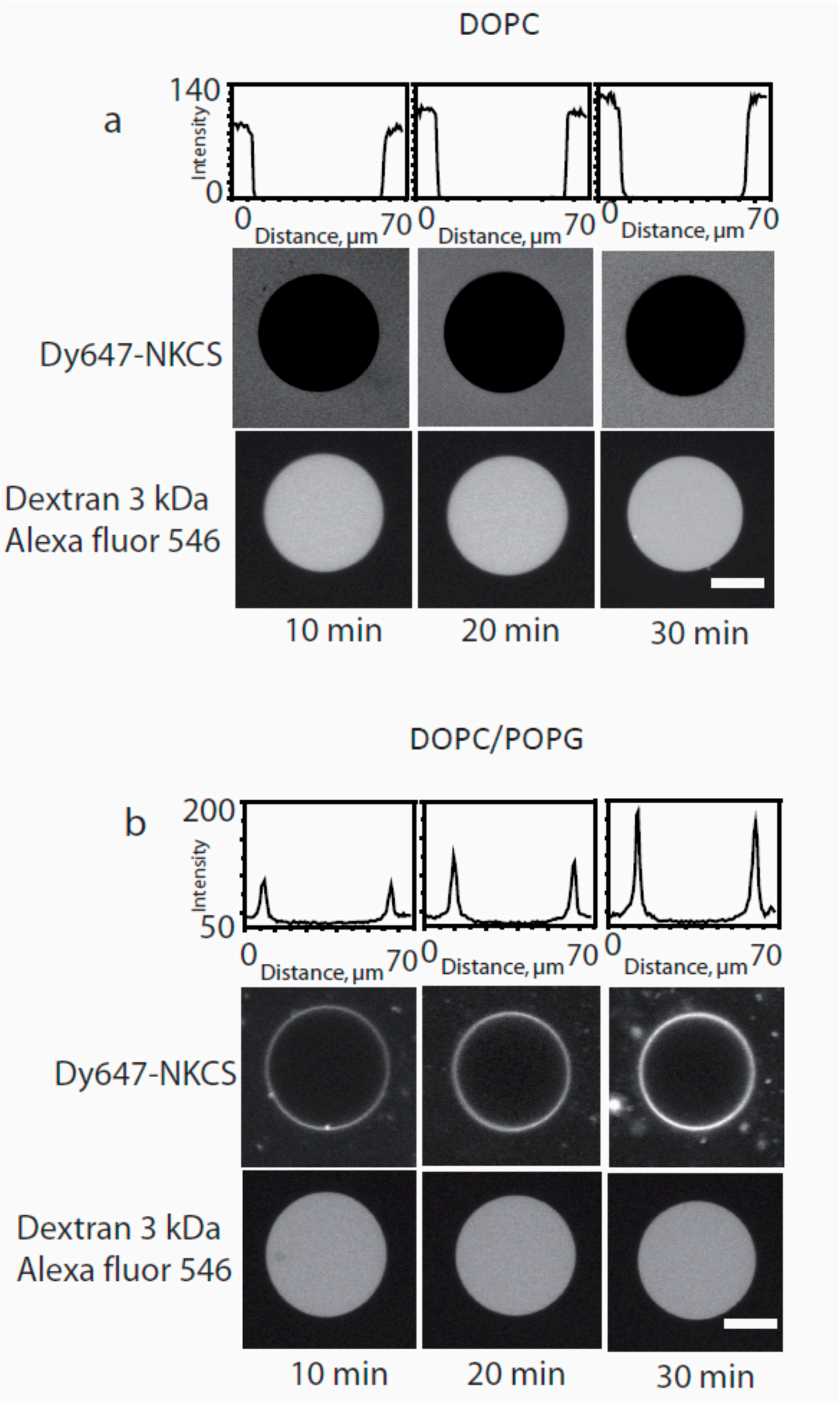

Figure S1. *Cont.*

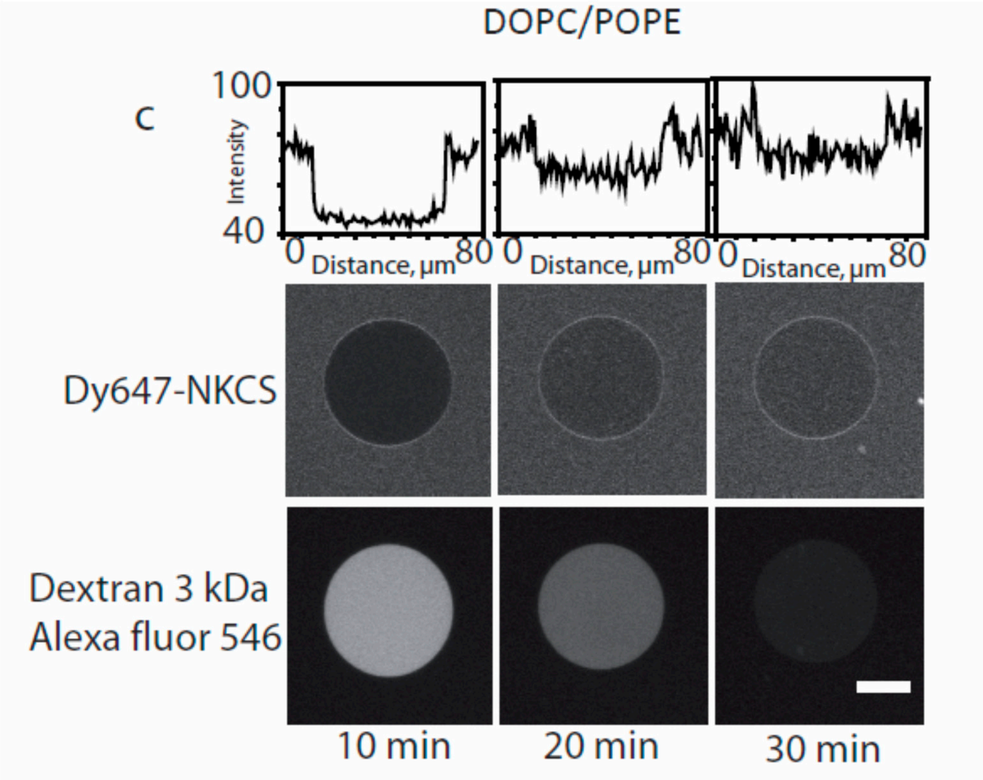

Figure S1. Translocation and tracer release induced by NKCS peptide in GUVs. (**a**) Interaction of NKCS with GUVs prepared from DOPC. The middle panel shows neither accumulation nor internalization of the peptide on the GUV membrane. The top panel quantifies the fluorescence intensity along a horizontal line through the center of the GUV. The lower panel demonstrates that 3 kDa dextran-Alexa Fluor 546 remained contained within the GUV; (**b**) The middle panel shows the binding the Dy647-NKCS to the membrane formed by DOPC/POPG. Peptide internalization was indicated by the non-zero fluorescence in the GUV interior, as quantified in the upper panel showing the fluorescence intensity along a horizontal line through the center of the GUV. For this GUV, peptide influx was very slow. The lower panel demonstrates that there was no leakage of 3 kDa dextran-Alexa Fluor 546 out of the GUV; (**c**) Interaction of NKCS with GUVs prepared from DOPC/DOPE. The top panel quantifies the fluorescence intensity along a horizontal line through the center of the GUV. The middle panel shows the accumulation of the peptide in the membrane. But additionally over time a portion of the NKCS peptide is crossing the membrane and can be found inside the versicle. This process was accompanied by the release of 3 kDa dextran labeled with Alexa Fluor 546 (lower panel).
